# Does Bupropion Impact More than Mood? A Case Report and Review of the Literature

**DOI:** 10.7759/cureus.4277

**Published:** 2019-03-19

**Authors:** Waqas Yasin, Syed Ijlal Ahmed, Robert V Gouthro

**Affiliations:** 1 Psychiatry, Medical College of Wisconsin, Green Bay, USA; 2 Internal Medicine, Liaquat National Hospital and Medical College, Karachi, PAK

**Keywords:** hypersexuality disorder, selective serotonin reuptake inhibitors (ssri), bupropion

## Abstract

Sexual side effects are often an unintended consequence of antidepressant treatment. Given antidepressants of different classes are frequently paired together in order to improve therapeutic response, understanding their interplay in regards to sexual functioning is important. Bupropion is an atypical antidepressant which is often used as a Selective Serotonin Reuptake Inhibitors adjunct in the treatment of depression, as well as in the amelioration of decreased libido. The following case highlights the importance of understanding antidepressant medications and their combined potential effect on sexual function to prevent unnecessary complications in cases involving the history of paraphilic disorders and hypersexuality.

## Introduction

Bupropion was first developed to improve the safety and tolerance of existing antidepressants [1]. It is associated with a unique clinical profile with efficacy comparable to that of other antidepressants. Devoid of clinically significant serotonergic effects or direct effects on postsynaptic receptors, bupropion is the only norepinephrine dopamine reuptake inhibitor (NDRI) currently approved by the Food and Drug Authority (FDA) for the treatment of depression. It is as effective as other antidepressants but is less likely to cause common antidepressant-associated side effects such as sexual dysfunction, weight gain, and sedation [2].

In addition to its similar effectiveness in the treatment of depression, it separates itself from many of the other available antidepressants by its activation, decrease in anhedonia via improvement of the impaired reward processing seen in depression [3], and superior sexual side effect profile which includes alleviation of sexual side effects induced by selective serotonin reuptake inhibitors (SSRIs) [4]. We are reporting a case of bupropion-induced hyper-sexuality when used as an adjunct to address anhedonia in a patient that presented with major depressive disorder and paraphilias. To the best of our knowledge, similar cases have rarely been reported in the medical literature [5].

## Case presentation

This is the case of a 57-year-old Caucasian male with a reported past history of major depressive disorder, adjustment disorder, anxiety disorder, and pedophilic disorder, referred by his primary care provider for “sad mood and irritability.”

The patient was released from prison two years prior to presentation after serving a sentence for first-degree sexual assault. This was his third incarceration for the sexual molestation of a child, the most recent being the three-year-old daughter of a family friend. There was no history of alcohol abuse or recreational drug use in our patient.

During incarceration in 2014, the patient was diagnosed with major depressive disorder and generalized anxiety disorder. This led to the initiation of fluoxetine which was eventually titrated to 60 mg daily and believed effective. In the fall of 2018, the patient informed his primary care physician of a progressive increase in anxiety, lack of motivation, low energy, and depressed mood, which began after his release from prison. The patient further relayed these symptoms complicated his adjustment to the outside world and led to social isolation. Both believed fluoxetine remained of partial benefit and bupropion was started at 150 mg daily as an adjunctive intervention to assist mood and anxiety.

Within weeks of this addition, the patient began to have an increase in sexual urges and fantasies. Both further affected his mood, anxiety, and level of isolation. After a referral to the resident mental health clinic, bupropion was discontinued and mirtazapine was initiated at 7.5 mg nightly as a replacement fluoxetine adjunct. Three weeks later, his sexual desires and urges were under better control and his mood and anxiety began to show significant improvements.

During the period between the cessation of bupropion and the improvement of elevated sexual desires, the patient reported improvement in social acceptance at his work. The reason for the decline in his active sexual urges was most likely due to the discontinuation of bupropion.

## Discussion

Review of the medical literature

Bupropion is effective for major depressive disorder (MDD) and seems to be a reasonable choice to address our patient’s mood symptoms [6]. Safety database entries for bupropion are extensive, comprising thousands of clinical trial subjects and including over 40 million patients who have received bupropion clinically [7]. Bupropion has been assessed for use in a number of on- and off-label indications given the uniqueness of its NDRI mechanism. It is believed its atypical mechanism of actions provides further benefit over SSRIs in its ability to improve anhedonia [3], fatigue, motivation, and focus [8], all were difficulties reported by our patient. Adjunctive use of bupropion with co-administered SSRIs is also not uncommon.

SSRIs are generally considered first-line agents for the treatment of depression, and a frequent side effect seen in their use is sexual dysfunction. Studies have reported this in 58% to 73% of men [9]. Thase et al. in their pooled SSRI comparator trial analysis concluded that bupropion was associated with less orgasmic dysfunction, sexual arousal disorder, and sexual desire disorder as compared to the SSRIs [10]. It is pivotal to note that, in this large data set, the risk of sexual dysfunction during bupropion therapy was virtually identical to that of placebo. Furthermore, a randomized controlled trial by Safarinejad concluded that SSRI-induced sexual dysfunction can effectively be reversed by a sustained released bupropion adjunct therapy in men [11].

A small double-blind study by Clayton et al. reported that bupropion, compared to placebo, increases the desire to engage in sexual activity and frequency of engaging in sexual activity [12-14]. A similar report by Borge et al. shows an increased libido as a bupropion side effect [15]. It is noteworthy that, in all of these studies, bupropion was used as a daily regimen.

Although it is not known if it was the original prescriber’s intention, it is believed that not only did fluoxetine use in our patient lead to improvement in mood and anxiety symptoms, it also assisted the patient by decreasing his sexual urges via libido suppression [16]. Fluoxetine’s sexual side effects can be used therapeutically in disorders involving hypersexuality and over arousal [17].

Our patient did notice an improvement in mood and motivation, but it is also theorized the addition of bupropion counteracted the suppression of sexual desires/fantasies by fluoxetine, and hence unmasked sexual urges which could have potentially led to serious consequences. It is further considered bupropion itself may have also led to an increase in libido and paraphilic fantasy. The increase in sexual focus seems unlikely to be related to mood improvement alone, as our patient had previously been stable in regards to mood while on fluoxetine monotherapy, and such a focus was not present.

Although providers of many specialties may be aware of the potential decrease in SSRI-related sexual side effects with the addition of adjunctive bupropion, we contend the negative impact of this reversal in patients with histories of hypersexuality or paraphilic disorders may not be readily apparent.

## Conclusions

We believe this case highlights the importance of understanding antidepressant medications and their combined potential effect on sexual function to prevent unnecessary complications in cases involving the history of paraphilic disorders and hypersexuality. More specifically, we conclude primary care and mental health providers should consider the possibility of elevated risk of hypersexuality in patients treated with SSRIs for major depressive disorder with the history of paraphilic disorders before the addition of bupropion as a depression treatment adjunct.
